# Sodium Intake and Incidence of Diabetes Complications in Elderly Patients with Type 2 Diabetes—Analysis of Data from the Japanese Elderly Diabetes Intervention Study (J-EDIT)

**DOI:** 10.3390/nu13020689

**Published:** 2021-02-21

**Authors:** Chika Horikawa, Rei Aida, Shiro Tanaka, Chiemi Kamada, Sachiko Tanaka, Yukio Yoshimura, Remi Kodera, Kazuya Fujihara, Ryo Kawasaki, Tatsumi Moriya, Hidetoshi Yamashita, Hideki Ito, Hirohito Sone, Atsushi Araki

**Affiliations:** 1 Department of Health and Nutrition, University of Niigata Prefecture Faculty of Human Life Studies, 471 Ebigase, Higashi-ku, Niigata 950-8680, Japan; horikawa@unii.ac.jp; 2 Department of Clinical Biostatistics, Graduate School of Medicine, Kyoto University, Yoshida-Konoe-cho, Sakyo-ku, Kyoto 606-8501, Japan; aida.rei.5c@kyoto-u.ac.jp (R.A.); tanaka.shiro.8n@kyoto-u.ac.jp (S.T.); 3 Training Department of Administrative Dietitians, Shikoku University, 123-1 Ebisuno, Furukawa, Ojin-cho, Tokushima 771-1151, Japan; c-kamada@shikoku-u.ac.jp (C.K.); yyoshimura@shikoku-u.ac.jp (Y.Y.); 4 Department of Public Health, Shiga University of Medical Science, Seta Tsukinowa-cho, Otsu, Sihga 520-2192, Japan; sachikot@belle.shiga-med.ac.jp; 5 Department of Endocrinology and Metabolism, Tokyo Metropolitan Geriatric Hospital, 35-2 Sakaecho, Itabashi-ku, Tokyo 173-0015, Japan; remi_kokubo@tmghig.jp (R.K.); hideki_ito@tmghig.jp (H.I.); 6 Department of Hematology, Endocrinology, and Metabolism, Niigata University Faculty of Medicine, 1-757 Asahimachi-dori, Chuoh-ku, Niigata 951-8510, Japan; kafujihara-dm@umin.ac.jp (K.F.); sone@med.niigata-u.ac.jp (H.S.); 7 Department of Vision Informatics, Graduate School of Medicine Faculty of Medicine, Osaka University, Osaka, 2-2 Yamadaoka, Suita 565-0871, Japan; ryo.kawasaki@ophthal.med.osaka-u.ac.jp; 8 Health Care Center, Kitasato University, 1-15-1, Kitazato, Minami-ku, Sagamihara-shi 252-0373, Japan; moriy@kitasato-u.ac.jp; 9 Department of Ophthalmology and Visual Science, Yamagata University Faculty of Medicine, 2-2-2 Iidanishi, Yamagata-shi 990-8560, Japan; yamasita@med.id.yamagata-u.ac.jp

**Keywords:** type 2 diabetes, older adults, medical nutritional therapy, sodium intake, diabetes complications

## Abstract

This study investigates the associations between sodium intake and diabetes complications in a nationwide cohort of elderly Japanese patients with type 2 diabetes aged 65–85. Data from 912 individuals regarding their dietary intake at baseline is analyzed and assessed by the Food Frequency Questionnaire based on food groups. Primary outcomes are times to diabetic retinopathy, overt nephropathy, cardiovascular disease (CVD), and all-cause mortality during six years. We find that mean sodium intake in quartiles ranges from 2.5 g to 5.9 g/day. After adjustment for confounders, no significant associations are observed between sodium intake quartiles and incidence of diabetes complications and mortality, except for a significant trend for an increased risk of diabetic retinopathy (*p* = 0.039). Among patients whose vegetable intake was less than the average of 268.7 g, hazard ratios (HRs) for diabetic retinopathy in patients in the second, third, and fourth quartiles of sodium intake compared with the first quartile were 0.87 (95% CI, 0.31–2.41), 2.61 (1.00–6.83), and 3.70 (1.37–10.02), respectively. Findings indicate that high sodium intake under conditions of low vegetable intake is associated with an elevated incidence of diabetic retinopathy in elderly patients with type 2 diabetes.

## 1. Introduction

Excessive sodium intake is regarded as a crucial factor related to the development and worsening of diabetes complications, and the importance of reducing dietary sodium intake is stated in guidelines for diabetes care [1,2,3]. However, epidemiological studies on the relationship between sodium intake and the development of diabetes complications are limited. Some previous prospective studies have examined the relationship between sodium intake and mortality in patients with type 1 diabetes [4] and type 2 diabetes—mainly among the middle-aged and young-old-aged [5,6,7], and results have been inconsistent. In a study of patients with type 1 diabetes, high 24-h urinary sodium excretion was related to the incidence of end-stage renal disease [4]. Our previous study as part of the Japan Diabetes Complications Study (JDCS) showed that high dietary sodium intake was associated with an elevated incidence of cardiovascular disease (CVD) in patients with type 2 diabetes aged 40–70 [6]. In Japan, there are two prospective longitudinal studies, namely, the JDCS mainly targeted toward middle-aged patients with type 2 diabetes [8,9], and the Japanese Elderly Diabetes Intervention Trial (J-EDIT) targeted to elderly patients with type 2 diabetes [10]. These two studies collected nearly the same information about anthropometric and laboratory tests, clinical characteristics, and outcomes for each patient [8,9,10].

The aging of the world’s population is a major contributor to the prevalence of diabetes [11]. According to the report from the International Diabetes Federation in 2019, 136 million people over 65 years old have diabetes [12]. The world’s population in 2019 was 7713 million, with 701 million over the age of 65 years [13]; thus, it is estimated that 1 in 5 elderly people have diabetes. Moreover, it is estimated that the percentage of the population over age 65 years could increase from 9.1% in 2019 to 15.9% in 2050 [13]. An increase in elderly patients with diabetes is inevitable. Thus, we investigated whether there was an association between sodium intake and the incidence of diabetes complications, including CVD, overt nephropathy, and diabetic retinopathy, as well as all-cause mortality in elderly patients with type 2 diabetes in the large nationwide multicenter cohort of J-EDIT.

## 2. Materials and Methods

### 2.1. Study Cohort

The study was conducted as part of J-EDIT, a multicenter prospective study on the development of macro- and microvascular complications and physical, psychological, and mental prognoses among Japanese patients with type 2 diabetes from outpatient departments in 39 representative hospitals. Eligible for the study were patients with type 2 diabetes aged 65–85 and HbA1c ≥7.9% or ≥7.4% with at least one abnormal finding among body mass index (BMI), blood pressure, or serum lipids. HbA1c assays were standardized by the Laboratory Test Committee of the Japan Diabetes Society (JDS) [3], and the National Glycohemoglobin Standardization Program value for HbA1c [14] was calculated by the following formula: 0.25 + 1.02 × JDS value [15]. A history of myocardial infarction, stroke, cancer, acute or serious illness, aphasia, and severe dementia within six months from enrollment were criteria for exclusion from the study. Details of study design and baseline characteristics of the J-EDIT were described elsewhere [10]. The protocol for the study, which is in accordance with the Declaration of Helsinki and the Ethical Guidelines for Clinical/Epidemiological Studies of the Japanese Ministry of Health Labor and Welfare, received ethical approval from the institutional review boards of all participating institutes. Written informed consent was obtained from all enrolled patients.

A total of 1173 patients who met the eligibility criteria described above were enrolled in the J-EDIT from March 2001 to February 2002, and 912 patients responded to a baseline dietary survey. The follow-up time was six years, and the dropout rate after six years was 8.9% (104 cases) [10]. In the present study, we grouped patients analyzed according to the following endpoints: CVD, nephropathy, and retinopathy. The CVD group consisted of 651 patients without a history of angina pectoris, myocardial infarction, or stroke. The nephropathy group included 624 patients after those with a baseline albumin excretion rate of <150 mg/g creatinine or serum creatine level >120 μmol/L were excluded. The retinopathy group was comprised of 327 patients after patients were excluded who had retinopathy or a major ocular disease (e.g., glaucoma, cataract, or a history of cataract surgery or vitrectomy).

### 2.2. Dietary Assessment

The Food Frequency Questionnaire based on food groups (FFQg) at baseline was used to assess nutritional and food intakes. The FFQg elicited information from the study participant on the average intake per week of 29 food groups and 10 kinds of cookery in commonly used units or portion sizes. The completed questionnaire was reviewed by a dietitian with the participant. The FFQg had been externally validated by comparison with dietary records for seven continuous days by 66 individuals aged 19–60 [16]. The average ratio of the estimates obtained by the FFQg against those by the dietary records was 104%, with ratios ranging from 72% to 121%. The correlation coefficient between the FFQg and dietary records for sodium intake was 0.43. To calculate the nutrient and food intakes, we used standardized software for population-based surveys and nutrition counseling in Japan (EIYO-KUN v.4.5, manufactured at the site of the Shikoku University Nutrition Database) based on Standard Tables of Food Composition in Japan [17] edited by the Japanese Ministry of Education, Culture, Sports, Science, and Technology.

### 2.3. Outcome Measures

Macroangiopathy endpoints included definite coronary heart disease (angina pectoris or myocardial infarction) or stroke. Coronary heart disease was diagnosed according to criteria defined by the Multinational Monitoring of Trends and Determinants in Cardiovascular Disease (WHO/MONICA) project [18,19]. Stroke was defined based on clinical signs of a focal neurological deficit with rapid onset persisting ≥24 h and confirmed by either computed tomography, or magnetic resonance imaging of the brain [20,21]. Confirmation of endpoints was made by a central committee comprised of experts in each complication based on additional data such as a detailed history, sequential changes in ECG and serum cardiac biomarkers, and results of coronary angiography or brain imaging. The endpoint of nephropathy was decided upon according to the development of overt nephropathy (spot urinary albumin excretion >300 mg/g creatinine in two consecutive samples). Japanese Diabetes Complication Study method was used to determine the presence of retinopathy, which was classified into five stages: Stage 0—no retinopathy; stage 1—dot hemorrhages, hemorrhages or hard exudates; stage 2—soft exudates; stage 3—intraretinal microvascular abnormalities or venous deformities; and stage 4—neovascularization, preretinal proliferative tissues, vitreous hemorrhages or retinal detachment. Development of retinopathy (from Stage 0 in both eyes at the time of registration to any other stage confirmed) in either eye indicated the endpoint of retinopathy. All-cause mortality was an additional endpoint. Details of the definition of each diabetes complication and all-cause mortality were previously published [10].

### 2.4. Statistical Analysis

Patient characteristics were described according to mean ± SD, median, interquartile range, or percentage. Univariate and multivariate Cox regression analyses were used to estimate the adjusted hazard ratios (HRs) and 95% Confidence Interval (CI) for each outcome after a six-year follow-up in relation to sodium intake at baseline by conducting a quartile analysis and assigning the lowest quartile of sodium intake as the referent. Multivariate adjusted analyses were conducted with adjustments for age, sex, BMI, HbA1c, diabetes duration, LDL-cholesterol, HDL-cholesterol, log-transformed triglycerides, insulin treatment, current smoker, alcohol intake, energy intake, physical activity, and systolic blood pressure (SBP). We also performed further analyses adjusted for the use of angiotensin II receptor blocker (ARB), angiotensin-converting enzyme (ACE) inhibitors, and calcium channel blockers at baseline in addition to the aforementioned model. Subgroup analyses in the Cox regression analysis were performed using the following groups: Sex (men vs. women), age (<75 years, ≥75 years), duration of diabetes (whether less than mean value or not), HbA1c (<8.5% vs. ≥8.5%), SBP (<140 mmHg vs. ≥140 mmHg), protein intake (whether < mean value or not) and vegetable intake (whether < mean value or not). All *p*-values are two-sided, and the significance level is 0.05. All statistical analyses and data management were conducted at a central data center using SAS ver. 9.4 (SAS Institute Inc., Cary, NC, USA).

## 3. Results

The baseline clinical characteristics of the 912 elderly patients according to quartiles of total sodium intake are shown in Table 1. Mean sodium intake was 4.1 g/day. Patients were divided into four groups according to quartiles of mean intakes of sodium as follows: Q1, 2.5 g/day; Q2, 3.5 g/day; Q3, 4.4 g/day; and Q4, 5.9 g/day. There were no significant differences in BMI, HbA1c, fasting blood glucose, serum lipids, serum creatinine, eGFR, systolic blood pressure, and prevalence of use of oral hypoglycemic agents, antihypertensive agents, and lipid-lowering agents among the quartiles of sodium intake. Patients in the highest quartile of sodium intake had the lowest prevalence of insulin treatment. At baseline, the prevalence of retinopathy was the lowest in those with the highest quartile of sodium intake (Q1, 44.7%; Q2, 45.6%; Q3, 42.1%; and Q4, 33.8%, respectively, trend *p* = 0.013).

Table 2 shows baseline daily dietary intake by the study patients with type 2 diabetes according to quartiles of sodium intake. Increases in total energy intake were observed from the first to the fourth quartile groups of sodium intake (Q1, 1425.2 kcal; Q2, 1647.3 kcal; Q3, 1796.1 kcal; and Q4, 2033.3 kcal; *p* < 0.001). The proportions of fat and protein as percentages of the energy supply in the higher sodium intake quartiles (Q2, Q3, and Q4) were significantly increased compared with the lowest sodium intake quartile (Q1), while the proportion of carbohydrates as the percentage of the energy supply were significantly decreased in the Q2, Q3, and Q4 quartiles (all *p* < 0.001). Patients with higher quartiles of sodium intake had significantly greater intakes of vitamins, minerals, and each food group.

During the six-year follow-up, in those in the first to the fourth quartiles of sodium intake there were 28, 17, 29, and 42 incidents of diabetic retinopathy, 1, 5, 7, and 4 incidents of overt nephropathy, and 17, 12, 11, and 12 incidents of CVD, respectively. Deaths according to the first to the fourth quartiles of sodium intake were 28, 19, 16, and 19, respectively. Table 3 shows HRs for sodium intake estimated by Cox regression models unadjusted (top model), adjusted for risk factors (middle model), and further adjusted for the use of ARB, ACE inhibitors, and calcium channel blockers at baseline (bottom model). In confounder-adjusted Cox regression, HRs and 95% CI for diabetic complications in patients in the second, third, and fourth quartiles of sodium intake were calculated using those in the first quartile of sodium intake as the reference. There were no significant associations between sodium intake quartiles and incidence of diabetes complications and mortality under the fully adjusted model (diabetic retinopathy: 0.92 (95%CI, 0.46–1.84), *p* = 0.802, 1.52 (0.79–2.92), *p* = 0.214, and 1.72 (0.91–3.24), *p* = 0.096; overt nephropathy: 3.33 (0.35–31.76), *p* = 0.296, 3.95 (0.40–38.93), *p* = 0.24, and 2.16 (0.17–27.12), *p* = 0.552; CVD: 0.92 (0.38–2.22), *p* = 0.846, 0.76 (0.30–1.94), *p* = 0.573, and 1.21 (0.46–3.21), *p* = 0.703) and mortality: 0.80 (0.38–1.66), *p* = 0.54, 0.80 (0.37–1.73), *p* = 0.565, and 1.03 (0.44–2.44), *p* = 0.948). However, a significant positive linear trend was shown between sodium intake and the risk of diabetic retinopathy (trend *p* = 0.039). There was no significant linear trend between sodium intake and the risk of overt nephropathy, CVD, and mortality (trend *p* = 0.729, 0.905, and 0.460, respectively).

The results of subgroup analysis of quartiles of sodium consumption and the incidence of diabetic retinopathy according to gender, age, duration of diabetes, HbA1c, SBP, protein intake, and vegetable intake are shown in Table 4. Subgroup analyses according to gender (men vs. women), age (<75 years, ≥75 years), duration of diabetes (whether < mean value (16.4 years) or not), HbA1c (<8.5% vs. ≥8.5%), SBP (<140 mmHg vs. ≥140 mmHg), and protein intake (whether < mean value (66.9 g) or not) did not reveal significant associations between quartiles of sodium intake and incident diabetic retinopathy. Among patients whose vegetable intake was <268.7 g/day, patients in the highest and second-highest quartiles (Q4 and Q3) of sodium intake had a significantly higher incidence of diabetic retinopathy compared with the first quartile (Q2: 0.87 (0.31–2.41), *p* = 0.785; Q3: 2.61 (1.00–6.83), *p* = 0.050; and Q4: 3.70 (1.37–10.02), *p* = 0.010). However, there was no significant differences between quartiles of sodium intake when the analysis was restricted to patients whose vegetable intake was ≥268.7 g/day (equal to or higher than participants’ mean vegetable intake) (Q2: 1.57 (0.45–5.44), *p* = 0.478; Q3: 1.91 (0.56–6.55), *p* = 0.306; and Q4: 1.51 (0.48–4.72), *p* = 0.483). As for a linear trend, a significant positive trend was shown between sodium intake and the risk of diabetic retinopathy in patients younger than 75 years, whose diabetes duration was lower than the average of 16.4 years, or whose vegetable intake was less than 268.7 g/day (trend *p* = 0.025, 0.028, 0.049, respectively). There was no significant linear trend between sodium intake and the risk of diabetic retinopathy in other patient groups.

## 4. Discussion

Guidelines for diabetes care [1,2,3] recommend a reduction in dietary sodium intake; however, there is no detailed evidence regarding the relationship between dietary sodium intake and the incidence of diabetes complications in elderly patients with type 2 diabetes. This six-year follow-up study of elderly Japanese patients with type 2 diabetes showed no significant associations of sodium intake with diabetic retinopathy, overt nephropathy, CVD, and all-cause mortality. However, high sodium intake under conditions of low vegetable intake was associated with an elevated incidence of diabetic retinopathy.

Our previous study based on data from the JDCS, which was mainly targeted toward middle-aged patients with type 2 diabetes, showed a significant relationship between high sodium intake and the incidence of CVD, although no such significant associations of high sodium intake with other diabetes complications and mortality were shown [6]. In that study, mean intakes of sodium by JDCS patients were Q1, 2.8 g/day; Q2, 3.8 g/day; Q3, 4.5 g/day; and Q4, 5.9 g/day. Those values were comparable to sodium intake by quartiles in the current J-EDIT study (Q1, 2.5 g/day; Q2, 3.5 g/day; Q3, 4.4 g/day; and Q4, 5.9 g/day). Current goals of daily dietary sodium intake for diabetes care are below 2.3 g/day in the guidelines of the American Diabetes Association (ADA) [1], 6 g/day according to the European Association for the Study of Diabetes (EASD) guidelines [2], and JDS recommends 7.5 g/day and 6.5 g/day salt intake for men and women, respectively, and 6 g/day for those with hypertension [3]. Comparing these guidelines with the lowest quartile of sodium consumption by J-EDIT patients, the J-EDIT patients consumed more sodium compared with that recommended by ADA and EASD guidelines and JDS guidelines for hypertensive patients with diabetes; however, intake was lower compared with JDS among normotensive men and women. The J-EDIT patients (3.8 g/day) consumed more sodium than was reported for USA and UK general populations (3.6 g/day and 3.4 g/day, respectively) [22], and a cohort of diabetes patients in the US (2.5–3.4 g/day) [23]. However, consumption of sodium among the JDCS patients was lower than in the general Japanese population in 2001 (4.5 g/day) [24]. Further studies are needed to clarify whether the medical nutritional treatment that restricts sodium intake to values according to the USA and European guidelines would reduce incident CVD in the management of diabetes.

Several plausible findings suggest the reasons why diabetes complications develop regardless of sodium intake in elderly patients with type 2 diabetes. First, the relationship between sodium intake and the risk of arteriosclerosis is not linear. A previous prospective study in 17 countries reported that mortality and cardiovascular events are greater on both a high sodium excretion, as well as on a low sodium excretion [25], and some populations demonstrate an increase in blood pressure on a low sodium diet and are considered to be inverse salt sensitive [26]. Therefore, some patients with diabetes who are inverse salt-sensitive and follow national federal guidelines for lower sodium consumption may have elevated blood pressure by activation of renin-aldosterone and sympathetic nerve systems [27], leading to the development of vascular complications. Second, elderly type 2 diabetes patients with low sodium intake have lower intakes of food groups and nutrition that were reported to be associated with reduced risk of diabetic complications and mortality. Our current study showed that study participants with low sodium intake had a significantly lower intake of all food groups and nutritional than those with high sodium intake. Several previous prospective studies reported that higher intakes of polyunsaturated fatty acids (PUFA), carotene, vitamin B6, and vitamin C were associated with a lower risk of developing retinopathy in patients with diabetes [28,29,30]. This suggests that low intake of those nutrients, as well as foods related to these nutrients, is associated with an increased risk of retinopathy in the low sodium intake quartile. This may have counteracted the effect of low sodium intake in preventing the development of atherosclerosis via blood pressure control.

In addition, in our subgroup analysis, the trend for risk of developing retinopathy at six years was positively correlated with increasing sodium intake at baseline in patients with type 2 diabetes younger than 75 years, but there was no such trend in those older than 75 years. Therefore, the impact of nutrients on health outcomes may vary with age [31]. It has been reported that the risk of death is increased in those with type 2 diabetes older than 75 years who have low protein, vegetable, and fish intakes [32,33]. Elderly patients with type 2 diabetes should have their dietary intake monitored not only for excessive sodium intake, but also for intakes other food groups and nutrition intakes that occur with low sodium intake to avoid dietary habits that would lead to malnutrition, sarcopenia, frailty development of diabetic complications and death.

Aging is a major risk factor that can have a significant impact on advanced glycation and progression of atherosclerosis, which are the main causes of the development of diabetes complications. The main risk factor profiles of advanced glycation end products and atherosclerosis-mediated diabetes complications include sex, age, diabetes-specific and glycemia-related factors, including HbA1c, diabetes duration, BMI, systolic blood pressure, lipid levels, and current smoking [34,35,36,37]. These can result from a complex combination of pathological pathways, such as hyperglycemia itself, increased insulin resistance, inflammatory responses, and decreased fluidity of the cell membrane [38]. Excessive sodium intake leads to chronic hypertension, a major cause of atherosclerosis [39,40]. It was reported that the administration of salt induced significant insulin resistance through the production of oxidative stress in salt-sensitive hypertensive rats [41,42], and that high sodium intake predicted the risk of type 2 diabetes in a prospective cohort study [43]. Since our current study enrolled elderly patients with type 2 diabetes, it can be inferred that insulin resistance and atherosclerosis, due to aging greatly affected the development of diabetes complications in the participants in J-EDIT, and the effect of sodium intake on insulin resistance and atherosclerosis was slight, unlike that in the middle-aged and young-old patients with type 2 diabetes reported in the JDCS [6].

As to the subgroup analysis according to vegetable intake, among patients whose vegetable intake was less than the average of 268.7 g, the HR for diabetic retinopathy in the top vs. the bottom quartile of sodium intake was significantly increased (3.70 (1.37–10.02), *p* = 0.010), but no significant association between sodium intake and diabetic retinopathy was observed in patients whose vegetable intake was equal to or higher than the participants’ mean vegetable intake. As to the reason for the various results for vegetable intake, low vegetable intake contributes to an increased risk of developing diabetic retinopathy. Increased fruit and vegetable intake was associated with reduced incident diabetic retinopathy among patients with type 2 diabetes [44]. Additionally, it was shown that the risk for diabetic retinopathy declined with increased intake of vitamin C and carotene, which are found in vegetables [17,44]. It can be speculated that the combination of low vegetable intake and high salt intake increased the risk of developing retinopathy in elderly patients with type 2 diabetes.

To the best of our knowledge, this is the first study on dietary sodium intake and the incidence of diabetes complications in which elderly patients with type 2 diabetes were prospectively registered based on HbA1c levels and not retrospectively selected based on self-reported diabetes status. Other strengths include treatment and follow-up plans that were conducted in institutes specializing in diabetes care and adjudication of cardiovascular events by an independent central committee.

The limitations of this study must be considered. First, the number of participants of our current study is limited to 912 though this study is conducted as a part of nationwide multicenter prospective study. In addition, this study investigated the association between sodium intake and diabetes complications in elderly patients with type 2 diabetes who had not developed any complications at the age of 65–85 years. J-EDIT previously reported that the proportion of elderly patients with type 2 diabetes with ischemic heart disease, cerebrovascular disease, retinopathy, and microalbuminuria or persistent proteinuria were 16%, 13%, 47%, and 43%, respectively [10]. Further studies are needed to examine the relationship between sodium intake and the severity and recurrence of diabetes complications in elderly patients with type 2 diabetes who had a history of diabetes complications. Second, the potential for bias, such as measurement errors in dietary assessments, confounding factors, and informative censoring, cannot be ruled out entirely. Third, as an observational study rather than a randomized trial, we could not conclude cause-effect relationships as to whether the medical nutritional treatment encouraging sodium reduction would reduce incident diabetic retinopathy in clinical practice. Moreover, it is not possible to say that sodium restriction is ineffective in elderly patients with type 2 diabetes. Fourth, our study did not observe any significant differences between blood pressure and dietary sodium intake. The percentage of patients treated by antihypertensive agents was similar in each quartile of dietary sodium intake. Given these results, a possible explanation may be that chronic high blood pressure could have been compensated for by increasing doses of antihypertensive drugs. Finally, our results may not be generally applicable to populations with different lifestyles or genetic factors. For example, the JDCS patients consumed a “high-carbohydrate low-fat” diet compared with Western patients with diabetes [45] and dietary sodium consumption in Japanese was generally higher than in Western people [22,23,24,45]. In addition, BMI and body weight are markedly different between patients in Japan and Western countries [46], and Asian patients have a much lower risk of CVD compared with Western patients and a higher risk of end-stage renal disease [47]. The contribution of such ethnic differences remains uncertain and is worthy of further research.

In conclusion, we found that high dietary sodium intake was not associated with the incidence of diabetic retinopathy, overt nephropathy, CVD, and all-cause mortality in elderly Japanese patients with type 2 diabetes. When these participants had a low vegetable intake, high sodium intake was associated with an elevated incidence of diabetic retinopathy. It was suggested that dietary salt restriction as a medical nutritional treatment for elderly patients with type 2 diabetes would be useful if consumption of vegetables is concomitantly encouraged.

## Figures and Tables

**Table 1:** Baseline clinical characteristics of the 912 elderly patients with type 2 diabetes according to quartiles of sodium intake.

	Q1 (*n* = 228)				Q2 (*n* = 228)				Q3 (*n* = 228)				Q4 (*n* = 228)				Trend
	*n*	Mean	±	SD	*n*	Mean	±	SD	*n*	Mean	±	SD	*n*	Mean	±	SD	*p*
Sodium intake (g/day)	228	2.5	±	0.5	228	3.5	±	0.2	228	4.4	±	0.3	228	5.9	±	0.9	<0.001
Age (y)	223	71.6	±	4.7	228	72.3	±	4.8	228	71.8	±	4.8	228	71.6	±	4.3	0.591
Women (%)	223	60.1	%		228	59.7	%		228	50.9	%		228	47.4	%		0.002
Diabetes duration (y)	221	17.8	±	9.6	222	15.8	±	9.8	223	16.2	±	9.3	227	15.8	±	9.4	0.053
Body mass index (kg/m^2^)	227	23.9	±	3.3	225	23.7	±	3.6	226	23.8	±	3.3	228	24.2	±	3.6	0.242
HbA1c (% NGSP value)	228	8.5	±	1.0	228	8.4	±	0.9	228	8.4	±	0.9	228	8.4	±	0.9	0.303
Fasting blood glucose (mg/dl)	201	171.4	±	55.6	201	160.2	±	43.7	211	171.8	±	53.0	211	166.3	±	47.2	0.825
Total cholesterol (mg/dl)	228	203.5	±	34.9	228	203.6	±	37.1	228	201.5	±	36.6	228	202.3	±	32.5	0.591
Triglycerides (mg/dl)	228	141.0	±	78.8	228	134.7	±	129.5	228	128.6	±	64.6	228	129.8	±	70.4	0.137
HDL cholesterol (mg/dl)	228	54.6	±	17.2	225	57.1	±	19.4	227	57.3	±	20.5	226	56.3	±	15.4	0.326
LDL cholesterol (mg/dl)	225	121.0	±	32.2	221	120.9	±	32.4	225	119.5	±	31.4	224	120.3	±	28.2	0.704
Systolic blood pressure (mmHg)	228	135.7	±	15.3	228	138.1	±	15.4	228	137.0	±	16.4	228	135.0	±	16.2	0.511
Diastolic blood pressure (mmHg)	228	74.0	±	9.4	228	75.3	±	10.4	228	75.5	±	9.3	227	74.7	±	9.5	0.417
Serum creatinine (mg/dl)	227	0.8	±	0.3	226	0.8	±	0.4	225	0.9	±	0.5	228	0.8	±	0.2	0.546
eGFR (mL/min/1.73m^2^)	226	65.3	±	20.3	226	65.0	±	18.9	224	66.3	±	19.1	228	67.4	±	18.3	0.195
Treatment																	
Treated by insulin (%)	227	37.0	%		226	27.9	%		226	28.3	%		228	27.6	%		0.043
Treated by OHA (%)	226	72.6	%		227	75.8	%		226	72.1	%		228	74.6	%		0.858
Treated by antihypertensives (%)	226	58.4	%		225	58.7	%		224	56.3	%		228	55.3	%		0.420
Treated by ACE inhibitor (%)	226	22.1	%		225	24.9	%		224	19.2	%		228	20.2	%		0.347
Treated by ARB (%)	226	9.7	%		225	8.0	%		224	7.6	%		228	11.4	%		0.588
Treated by calcium channel blockers (%)	226	44.7	%		225	44.0	%		224	42.0	%		228	40.4	%		0.306
Treated by lipid-lowering agents (%)	226	42.0	%		227	40.1	%		225	41.8	%		228	35.1	%		0.186
Treated by antiplatelet agents (%)	227	29.1	%		227	25.1	%		224	31.7	%		228	25.0	%		0.667
Treated by anticoagulants (%)	226	3.1	%		226	2.7	%		225	1.8	%		226	1.8	%		0.282
History of complications																	
Cardiovascular disease (%)	228	28.1	%		228	30.7	%		228	33.8	%		228	20.6	%		0.148
Nephropathy (%)	209	18.2	%		215	13.0	%		213	15.5	%		216	13.9	%		0.350
Retinopathy (%)	228	44.7	%		228	45.6	%		228	42.1	%		228	33.8	%		0.013
Leisure-time physical activity (METs)	198	12.1	±	18.9	189	15.1	±	27.9	195	15.2	±	28.5	189	15.4	±	26.9	0.214
Current smoker (%)	213	14.6	%		214	14.5	%		214	14.0	%		219	18.7	%		0.270
Current alcohol drinker (%)	211	24.2	%		214	29.9	%		214	31.3	%		219	30.1	%		0.167

Abbreviations: ACE, angiotensin-converting enzyme; ARB, angiotensin II receptor blocker; eGFR, estimated glomerular filtration rate; OHA, oral hypoglycemic agent.

**Table 2:** Baseline food groups and nutritional intakes of the 912 elderly patients with type 2 diabetes according to quartiles of sodium intake.

	Q1 (*n* = 228)			Q2 (*n* = 228)			Q3 (*n* = 228)			Q4 (*n* = 228)			Trend
	Mean	±	SD	Mean	±	SD	Mean	±	SD	Mean	±	SD	*p*
Sodium intake (g/day)	2.5	±	0.5	3.5	±	0.2	4.4	±	0.3	5.9	±	0.9	<0.001
Nutritional intake													
Energy (kcal/day)	1425.2	±	240.6	1647.3	±	261.3	1796.1	±	296.3	2033.3	±	379.2	<0.001
Protein (g/day)	52.7	±	13.2	63.2	±	13.2	70.7	±	15.7	81.2	±	19.6	<0.001
Protein (% energy)	14.7	±	2.2	15.4	±	2.1	15.7	±	2.0	16.0	±	2.2	<0.001
Fat (g/day)	37.7	±	11.6	47.1	±	12.5	52.5	±	13.8	62.4	±	20.1	<0.001
Fat (% energy)	23.5	±	4.7	25.6	±	4.4	26.1	±	4.1	27.2	±	4.9	<0.001
Carbohydrate (g/day)	210.7	±	30.6	233.1	±	37.5	250.2	±	38.5	276.7	±	47.6	<0.001
Carbohydrate (% energy)	61.8	±	6.0	59.1	±	5.5	58.2	±	5.3	56.8	±	5.8	<0.001
Fiber, total (g/day)	10.9	±	3.2	13.4	±	3.6	14.9	±	3.7	17.2	±	4.2	<0.001
Vitamin A (μg RAE/day)	803.4	±	362.7	993.4	±	404.6	1055.6	±	415.1	1187.1	±	433.8	<0.001
Vitamin D (μg/day)	7.1	±	3.6	9.1	±	3.7	10.5	±	4.4	12.4	±	5.4	<0.001
Vitamin E (mg/day)	5.8	±	1.5	7.3	±	1.7	8.0	±	1.7	9.4	±	2.2	<0.001
Vitamin K (mg/day)	187.1	±	75.0	234.0	±	84.8	251.2	±	88.3	291.1	±	96.1	<0.001
Vitamin B1 (mg/day)	0.7	±	0.2	0.8	±	0.2	0.9	±	0.2	1.1	±	0.3	<0.001
Vitamin B2 (mg/day)	0.8	±	0.2	1.0	±	0.2	1.1	±	0.3	1.3	±	0.3	<0.001
Niacin (mg/day)	11.5	±	3.8	14.1	±	3.9	16.0	±	4.5	18.6	±	5.7	<0.001
Vitamin B6 (mg/day)	1.0	±	0.3	1.2	±	0.3	1.3	±	0.3	1.5	±	0.4	<0.001
Vitamin B12 (μg/day)	6.0	±	2.9	7.5	±	3.0	8.8	±	3.7	10.4	±	4.5	<0.001
Folate (mg/day)	250.1	±	81.0	303.6	±	90.6	331.5	±	97.5	375.6	±	100.2	<0.001
Pantothenic acid (mg/day)	4.5	±	1.0	5.2	±	1.0	5.7	±	1.1	6.4	±	1.4	<0.001
Vitamin C (mg/day)	87.8	±	36.5	106.3	±	40.9	119.3	±	41.7	137.2	±	44.6	<0.001
Potassium (mg/day)	1926.8	±	515.0	2334.8	±	563.2	2636.5	±	585.1	3033.0	±	680.6	<0.001
Calcium (mg/day)	479.5	±	148.6	580.5	±	165.0	650.9	±	165.5	734.9	±	212.7	<0.001
Magnesium (mg/day)	209.3	±	49.0	251.5	±	55.7	278.5	±	55.8	323.0	±	69.3	<0.001
P (mg/day)	837.5	±	202.0	993.3	±	206.3	1106.8	±	229.0	1262.2	±	285.1	<0.001
Fe (mg/day)	6.6	±	1.5	8.0	±	1.8	8.8	±	1.9	10.2	±	2.3	<0.001
Zn (μg/day)	16.1	±	12.5	24.0	±	18.4	31.8	±	24.8	42.2	±	33.0	<0.001
Cu (mg/day)	1.4	±	0.6	1.9	±	0.9	2.3	±	1.2	3.0	±	1.6	<0.001
Mn (mg/day)	2.4	±	0.5	2.7	±	0.6	2.8	±	0.5	3.2	±	0.6	<0.001
Intake of food groups													
Grain (g/day)	192.3	±	29.1	196.7	±	32.2	197.8	±	33.7	204.2	±	40.1	<0.001
Potatoes (g/day)	30.7	±	28.3	34.8	±	24.8	47.4	±	32.9	50.3	±	33.1	<0.001
Vegetables (g/day)	206.519	±	107.6	258.824	±	116.6	285.662	±	132.1	323.675	±	128.7	<0.001
Fruits (g/day)	102.5	±	68.7	118.0	±	82.2	132.6	±	77.3	159.0	±	83.6	<0.001
Seaweed (g/day)	2.1	±	1.5	2.5	±	1.5	2.7	±	1.9	3.2	±	2.0	<0.001
Meat (g/day)	30.7	±	27.8	41.4	±	33.1	46.5	±	35.9	58.0	±	56.6	<0.001
Fish/Seafood (g/day)	62.6	±	38.4	78.8	±	41.3	94.6	±	50.1	112.0	±	60.0	<0.001
Eggs (g/day)	23.5	±	15.8	26.6	±	15.8	28.0	±	17.6	31.9	±	20.3	<0.001
Soybeans/Soy products (g/day)	55.7	±	36.2	67.5	±	39.8	73.9	±	41.6	86.3	±	47.8	<0.001
Milk/Dairy products (g/day)	140.8	±	80.3	151.5	±	89.1	171.5	±	94.0	175.9	±	126.8	<0.001
Snacks/Candies (g/day)	26.8	±	24.2	39.8	±	32.1	47.6	±	38.9	58.3	±	49.3	<0.001
Ethanol (g/day)	3.0	±	11.8	3.8	±	16.0	3.9	±	12.3	6.3	±	22.3	0.041

**Table 3:** Cox regression analysis of diabetes complications and all-cause mortality according to quartiles of sodium intake.

	Q1		Q2				Q3				Q4				Trend
	Events/Pts	HR	Events/Pts	HR	95%CI	*p*	Events/Pts	HR	95%CI	*p*	Events/Pts	HR	95%CI	*p*	*p*
Diabetic retinopathy (*n* = 327)															
Not adjusted	28/82	ref	17/65	0.72	0.39–1.31	0.276	29/75	0.94	0.56–1.58	0.820	42/105	1.04	0.65–1.69	0.861	0.202
Adjusted *	21/68	ref	14/53	0.96	0.48–1.92	0.904	24/63	1.32	0.69–2.49	0.401	36/83	1.66	0.88–3.13	0.115	0.059
Further Adjusted **	21/68	ref	14/53	0.92	0.46–1.84	0.802	24/62	1.52	0.79–2.92	0.214	36/83	1.72	0.91–3.24	0.096	0.039
Diabetic overt nephropathy (*n* = 624)															
Not adjusted	1/145	ref	5/169	3.62	0.40–32.34	0.250	7/158	6.36	0.78–51.67	0.084	4/152	3.90	0.44–34.85	0.224	0.233
Adjusted *	1/118	ref	4/133	3.97	0.41–38.23	0.233	7/133	4.80	0.50–46.04	0.174	4/121	2.37	0.19–30.01	0.505	0.671
Further Adjusted **	1/118	ref	4/133	3.33	0.35–31.76	0.296	7/133	3.95	0.40–38.93	0.240	4/121	2.16	0.17–27.12	0.552	0.729
Cardiovascular disease (*n* = 651)															
Not adjusted	17/162	ref	12/157	0.74	0.35–1.55	0.423	11/151	0.66	0.31–1.40	0.277	12/181	0.62	0.30–1.31	0.210	0.205
Adjusted *	14/137	ref	9/119	0.94	0.39–2.28	0.892	10/132	0.85	0.34–2.08	0.715	11/147	1.20	0.45–3.19	0.719	0.876
Further Adjusted **	14/136	ref	9/118	0.92	0.38–2.22	0.846	9/130	0.76	0.30–1.94	0.573	11/147	1.21	0.46–3.21	0.703	0.905
All-cause mortality (*n* = 912)															
Not adjusted	28/228	ref	15/228	0.63	0.33–1.17	0.143	19/228	0.76	0.42–1.37	0.368	16/228	0.62	0.33–1.15	0.131	0.092
Adjusted *	23/191	ref	13/178	0.79	0.38–1.64	0.530	14/191	0.94	0.45–1.97	0.865	13/184	1.02	0.43–2.40	0.964	0.510
Further Adjusted **	23/190	ref	13/177	0.80	0.38–1.66	0.540	12/189	0.80	0.37–1.73	0.565	13/184	1.03	0.44–2.44	0.948	0.460

* Adjusted for age, sex, BMI, HbA1c, diabetes duration, LDL-cholesterol, HDL-cholesterol, log-transformed triglycerides, treatment by insulin, current smoker, alcohol intake, energy intake, physical activity, and systolic blood pressure. ** Further adjusted for the use of ARB, ACE inhibitors, and calcium channel blockers at baseline.

**Table 4:** Subgroup analysis of quartile of sodium and incidence of diabetic retinopathy.

	Q1 (*n* = 82)		Q2 (*n* = 65)				Q3 (*n* = 75)				Q4 (*n* = 105)				Trend
	Events/Pts	HR	Events/Pts	HR	95%CI	*p*	Events/Pts	HR	95%CI	*p*	Events/Pts	HR	95%CI	*p*	*p*
Age <75 y (*n* = 237)															
Not adjusted	19/62	ref	14/42	1.06	0.51–2.18	0.880	24/56	1.30	0.70–2.42	0.413	31/77	1.41	0.78–2.55	0.262	0.173
Adjusted *	15/53	ref	12/36	1.33	0.60–2.93	0.484	20/46	1.75	0.84–3.63	0.136	26/61	1.97	0.93–4.19	0.077	0.048
Further Adjusted **	15/53	ref	12/36	1.24	0.56–2.73	0.602	20/46	2.13	0.99–4.56	0.052	26/61	2.12	1.00–4.50	0.050	0.025
Age ≥75 y (*n* = 90)															
Not adjusted	9/20	ref	3/23	0.30	0.08–1.14	0.077	5/19	0.32	0.09–1.11	0.072	11/28	0.72	0.29–1.81	0.490	0.859
Adjusted *	6/15	ref	2/17	0.06	0.01–0.70	0.024	4/17	0.13	0.02–1.11	0.062	10/22	0.29	0.03–2.46	0.255	0.436
Further Adjusted **	6/15	ref	2/17	0.08	0.01–0.84	0.035	4/16	0.20	0.02–2.10	0.180	10/22	0.48	0.04–5.79	0.564	0.426
Males (*n* = 156)															
Not adjusted	12/38	ref	9/27	1.03	0.43–2.50	0.948	18/38	1.15	0.54–2.47	0.714	17/53	0.89	0.42–1.92	0.772	0.782
Adjusted *	10/32	ref	7/22	1.19	0.42–3.37	0.742	15/31	1.76	0.73–4.27	0.211	14/43	1.32	0.53–3.30	0.557	0.285
Further Adjusted **	10/32	ref	7/22	1.10	0.37–3.24	0.864	15/31	1.84	0.74–4.53	0.187	14/43	1.36	0.53–3.45	0.521	0.214
Females (*n* = 171)															
Not adjusted	16/44	ref	8/38	0.59	0.24–1.45	0.248	11/37	0.80	0.36–1.76	0.572	25/52	1.53	0.79–2.94	0.205	0.135
Adjusted *	11/36	ref	7/31	0.67	0.24–1.93	0.462	9/32	0.84	0.30–2.39	0.745	22/40	1.58	0.60–4.15	0.353	0.253
Further Adjusted **	11/36	ref	7/31	0.66	0.23–1.91	0.441	9/31	0.91	0.30–2.76	0.861	22/40	1.68	0.61–4.63	0.315	0.276
Duration of diabetes <16.4 y (*n* = 221)															
Not adjusted	19/56	ref	11/42	0.73	0.34–1.57	0.420	19/55	0.87	0.44–1.68	0.668	29/68	1.28	0.69–2.35	0.434	0.212
Adjusted *	12/46	ref	9/38	0.93	0.37–2.30	0.869	16/47	1.09	0.49–2.41	0.833	26/57	1.78	0.81–3.93	0.152	0.037
Further Adjusted **	12/46	ref	9/38	0.94	0.38–2.36	0.897	16/47	1.15	0.51–2.59	0.745	26/57	1.85	0.83–4.12	0.131	0.028
Duration of diabetes ≥16.4 y (*n* = 101)															
Not adjusted	9/26	ref	6/20	1.23	0.40–3.76	0.719	10/19	1.56	0.60–4.05	0.363	13/36	1.10	0.46–2.66	0.833	0.665
Adjusted *	9/22	ref	5/15	1.95	0.47–8.11	0.360	8/16	4.26	1.00–18.16	0.050	10/26	2.69	0.72–10.01	0.140	0.534
Further Adjusted **	9/22	ref	5/15	2.32	0.49–10.88	0.287	8/15	5.79	1.12–30.11	0.037	10/26	2.95	0.80–10.97	0.106	0.404
HbA1c <8.5% (*n* = 213)															
Not adjusted	15/52	ref	9/42	0.88	0.36–2.17	0.783	17/48	1.05	0.50–2.22	0.898	28/71	1.61	0.81–3.18	0.174	0.099
Adjusted *	12/43	ref	7/33	0.98	0.37–2.61	0.967	12/38	1.10	0.47–2.58	0.835	24/55	2.20	0.94–5.16	0.070	0.075
Further Adjusted **	12/43	ref	7/33	0.95	0.36–2.54	0.922	12/37	1.14	0.47–2.78	0.768	24/55	2.22	0.93–5.29	0.073	0.074
HbA1c ≥8.5% (*n* = 114)															
Not adjusted	13/30	ref	8/23	0.66	0.27–1.59	0.351	12/27	1.06	0.48–2.33	0.894	14/34	0.83	0.39–1.77	0.633	0.974
Adjusted *	9/25	ref	7/20	0.77	0.27–2.18	0.619	12/25	0.76	0.22–2.63	0.666	12/28	0.69	0.22–2.21	0.535	0.681
Further Adjusted **	9/25	ref	7/20	0.59	0.19–1.82	0.361	12/25	0.83	0.25–2.82	0.769	12/28	0.51	0.15–1.79	0.295	0.683
Systolic blood pressure <140 mmHg (*n* = 195)															
Not adjusted	18/49	ref	12/38	0.93	0.43–2.01	0.850	14/44	0.70	0.34–1.45	0.335	28/64	1.31	0.71–2.44	0.390	0.408
Adjusted *	14/40	ref	10/31	1.12	0.48–2.64	0.792	11/36	0.98	0.41–2.37	0.966	23/51	1.76	0.74–4.20	0.202	0.319
Further Adjusted **	14/40	ref	10/31	1.07	0.45–2.53	0.885	11/36	1.17	0.47–2.89	0.734	23/51	1.93	0.81–4.65	0.140	0.221
Systolic blood pressure ≥140 mmHg (*n* = 132)															
Not adjusted	10/33	ref	5/27	0.60	0.20–1.79	0.360	15/31	1.55	0.67–3.56	0.302	14/41	1.07	0.47–2.48	0.868	0.317
Adjusted *	7/28	ref	4/22	0.76	0.21–2.76	0.681	13/27	2.22	0.78–6.34	0.136	13/32	1.46	0.46–4.60	0.521	0.193
Further Adjusted **	7/28	ref	4/22	0.74	0.20–2.70	0.646	13/26	2.81	0.88–9.01	0.083	13/32	1.84	0.54–6.33	0.333	0.230
Protein intake <66.9g/day (*n* = 136)															
Not adjusted	22/64	ref	12/42	0.77	0.37–1.61	0.488	14/37	1.05	0.53–2.08	0.891	8/23	0.95	0.42–2.17	0.911	0.808
Adjusted *	16/52	ref	9/34	0.91	0.37–2.19	0.825	12/30	1.37	0.57–3.30	0.477	7/17	0.93	0.35–2.47	0.877	0.426
Further Adjusted **	16/52	ref	9/34	0.83	0.34–2.04	0.683	12/30	1.81	0.69–4.75	0.227	7/17	1.32	0.45–3.87	0.611	0.238
Protein intake ≥66.9 g/day (*n* = 161)															
Not adjusted	6/18	ref	5/23	0.91	0.24–3.39	0.886	15/38	1.13	0.37–3.42	0.834	34/82	1.48	0.53–4.19	0.456	0.188
Adjusted *	5/16	ref	5/19	0.73	0.18–2.86	0.646	12/33	0.96	0.30–3.11	0.949	29/66	1.47	0.48–4.53	0.500	0.190
Further Adjusted **	5/16	ref	5/19	0.62	0.15–2.49	0.498	12/32	1.01	0.31–3.31	0.983	29/66	1.56	0.52–4.74	0.431	0.137
Vegetable intake <268.7 g/day (*n* = 188)															
Not adjusted	21/65	ref	7/37	0.61	0.26–1.44	0.258	16/47	0.90	0.46–1.76	0.765	15/39	1.18	0.60–2.32	0.638	0.400
Adjusted *	16/53	ref	6/32	0.96	0.35–2.64	0.934	14/41	1.81	0.75–4.34	0.185	14/31	2.68	1.07–6.70	0.035	0.075
Further Adjusted **	16/53	ref	6/32	0.87	0.31–2.41	0.785	14/40	2.61	1.00–6.83	0.050	14/31	3.70	1.37–10.02	0.010	0.049
Vegetable intake ≥268.7 g/day (*n* = 139)															
Not adjusted	7/17	ref	10/28	1.14	0.38–3.39	0.819	13/28	1.46	0.51–4.15	0.479	27/66	1.42	0.55–3.70	0.471	0.828
Adjusted *	5/15	ref	8/21	1.40	0.42–4.73	0.585	10/22	1.44	0.44–4.68	0.547	22/52	1.36	0.44–4.21	0.591	0.479
Further Adjusted **	5/15	ref	8/21	1.57	0.45–5.44	0.478	10/22	1.91	0.56–6.55	0.306	22/52	1.51	0.48–4.72	0.483	0.437

* Adjusted for age, sex, BMI, HbA1c, diabetes duration, LDL-cholesterol, HDL-cholesterol, log-transformed triglycerides, treatment by insulin, current smoker, alcohol intake, energy intake, physical activity, and systolic blood pressure. ** Further adjusted for the use of ARB, ACE inhibitors, and calcium channel blockers at baseline. In each subgroup analysis, the variable was not adjusted for.

## Data Availability

The data presented in this study are available on request from the corresponding author. The data are not publicly available due to privacy of participants.
